# Exploring classroom-based assessment for young EFL learners in the Chinese context: Teachers’ beliefs and practices

**DOI:** 10.3389/fpsyg.2022.1051728

**Published:** 2022-11-14

**Authors:** Qiaozhen Yan, Lawrence Jun Zhang, Helen R. Dixon

**Affiliations:** ^1^School of Foreign Languages and Culture, Chongqing University, Chongqing, China; ^2^Faculty of Education and Social Work, University of Auckland, Auckland, New Zealand

**Keywords:** classroom-based assessment, young language learners, teachers’ beliefs, Chinese English-as-a-foreign-language learners, teacher education, China

## Abstract

Classroom-based assessment (CBA) is an approach for learning improvement that has been advocated as having strong potential in enhancing learner autonomy of young language learners (YLLs). This study investigated Chinese primary school English as a foreign language (EFL) teachers’ beliefs about CBA, their assessment practices, and the relationship between their CBA beliefs and practices. Drawing on data from a survey of 195 Chinese primary school EFL teachers, results showed that the teachers positively believed in the value of various CBA processes, including planning assessment, collecting learning evidence, making professional judgments and providing appropriate feedback, and they also attempted to enact these assessment practices; belief-practice alignment was also identified, showing that teachers’ beliefs about CBA were significant predictors of their assessment practices. Implications are provided for promoting the implementation of CBA for YLLs in similar contexts.

## Introduction

Classroom-based assessment (CBA) emphasizes the integration of assessment in the instructional process in order to facilitate student learning (Cizek, 2010). Ever since Black and Wiliam (1998) landmark work on the role of teachers’ classroom assessment in maximizing students’ learning gains, CBA has been drawing increasing research attention. CBA is considered particularly critical for young language learners (YLLs), defined as children aged approximately 6–12 learning a foreign or second language (Britton, 2021). This is because YLLs bring to their language learning their own unique characteristics of cognitive, social, emotional and physical growth, literacy development, and vulnerability (McKay, 2006). Characteristics such as these warrant special attention to assessment of YLLs. Thus, CBA has been strongly promoted for YLLs given its potential to foster children’s development of self-regulation (Butler, 2021).

However, despite a growing research interest in assessment of YLLs, little empirical evidence has been reported on how language teachers implement CBA in young learner contexts. In their critical review of the most important publications on assessing YLLs’ language abilities over the past two decades, Nikolov and Timpe-Laughlin (2021) noted that CBA for YLLs is an area in need of more consideration and research. Specifically, the limited body of research on CBA for YLLs has examined the accuracy of CBA as a tool for measuring the language abilities of YLLs, effectiveness of CBA as a means of stimulating language learning of YLLs, and teachers’ CBA practices for YLLs as well as influencing factors (e.g., Butler and Lee, 2010; Liu and Brantmeier, 2019; Kaur, 2021). These studies yielded mixed results about the accuracy of CBA for YLLs, unpacked the effects of CBA on YLLs’ language learning performance and attitudes, and offered insights into the current status of the implementation of CBA for YLLs. Nevertheless, much less attention has been paid to teachers’ beliefs and practices pertinent to CBA for YLLs. This is an important gap because teachers’ beliefs can have a powerful impact on their instructional practices (Pajares, 1992; Borg, 2003; Dixon et al., 2011; Sun and Zhang, 2021). The extent to which CBA is effectively implemented in young learner classrooms will be affected by teachers’ beliefs about the purposes and nature of CBA. Examining the interaction between teachers’ CBA beliefs and practices, therefore, can help us better understand how CBA can be integrated into language instruction to young learners. Understanding teachers’ beliefs is also an essential part of teacher education programs that aim to promote change in teachers’ classroom behaviors (Borg, 2011; Wu et al., 2021a). Studying teachers’ beliefs about CBA, therefore, may shed light on how to design teacher professional development activities aimed at promoting the enactment of CBA for YLLs.

To fill this void, this study aims to investigate primary school English-as-a-foreign-language (EFL) teachers’ beliefs and practices related to CBA in the Chinese context. The study contributes to the literature by uncovering teacher belief-practice relationships in CBA for YLLs. It also seeks to offer practical implications for teacher educators, school administrators and policy makers so as to help advance the implementation of CBA in young EFL learner contexts.

## Literature review

### CBA

CBA refers to any assessment embedded in classroom instruction, either explicit or implicit, regarded as in opposition to traditional large-scale tests external to the classroom (Turner, 2012). Technically, it is viewed as a process that “teachers and students use in collecting, evaluating, and using evidence of student learning” (McMillan, 2013, p. 1). In this sense, CBA is a process-based practice rather than a simple assessment instrument. CBA is broadly conceived as serving two purposes: summative assessment (SA) and formative assessment (FA), often referred to as assessment of learning and assessment for learning, respectively (Brookhart, 2004; Wu et al., 2021c). SA is primarily designed to ‘elicit evidence regarding the amount or level of knowledge, expertise or ability’ (Wiliam, 2001, p. 169) for administrative or reporting purposes, with a focus on scores. FA, on the other hand, focuses on using assessment to promote student learning (Black and Wiliam, 2009). It emphasizes the provision of descriptive feedback (rather than scores) as evidence about student learning, through which students are informed about their strengths and weakness, and scaffolded to close the gap between their current and desired performance (Sadler, 1989). Students, in particular, play a vital role in the assessment process, whereby they have an awareness of learning goals and assessment criteria, and actively engage themselves in self-and peer assessment (Pryor and Crossouard, 2008). Assessment conducted in this way is seen to have great potential in developing students’ capacity to self-regulate their learning (Assessment Reform Group, 2002; Panadero et al., 2018). Notwithstanding the two different purposes for assessment, the boundary between SA and FA is not as clear cut as usually represented because the same assessment information can be used for different purposes at different times (Rea-Dickins, 2007). It is how the information is used which provides the key distinction. In some cases, SA can be used formatively and FA can be used to serve a summative function (Dixson and Worrell, 2016; Dolin et al., 2018). Summative tests, for example, which typically take place at the end of a unit or term to record learning attainments, can be used to adjust teaching and improve students’ learning in the future. FA practices, such as self-and peer assessment, have the potential to generate a lot of data about students’ progress, which can be recorded and further used for summative reporting purposes. More recently, scholars have pointed out that, in an assessment for learning culture, all assessments, including those for administrative and reporting purposes, can and need to be implemented with a central aim of facilitating learning (Black and Wiliam, 2018; Davison, 2019).

Researchers have put forward a number of frameworks casting light on how to translate CBA for learning principles into practice. These include Black and Wiliam (2009) five strategies of formative assessment, Hill and McNamara (2011) framework for CBA processes, Davison and Leung (2009) framework for teachers-based assessment, and more recently learning-oriented assessment related frameworks (Carless, 2011; Turner and Purpura, 2016). Despite their differences, all the frameworks emphasize three instructional processes that are critical to student learning improvement, i.e., where learners are going, where they are in their learning, and how to get there (Wiliam and Thompson, 2008). Davison and Leung (2009) framework is particularly operational as it conceptualizes CBA into a cycle of four steps, addressing the process-based feature of CBA. It provides a working approach for the analysis of teachers’ classroom-embedded assessment practice, and thus was selected as the analytical framework for the present study.

The first step of Davison and Leung (2009) framework, planning assessment (PA), focuses on clarifying learning goals and assessment criteria to ensure that students have an awareness of where they are going. Selecting assessment methods that suit the needs of students is also an important element of this step. The second step, collecting learning evidence, is to collect instructionally tractable evidence of student learning, which can be achieved through various methods, such as spontaneous assessment opportunities (SAO), planned assessment opportunities, and formal assessment tasks (FATs; Hill and McNamara, 2011; Turner and Purpura, 2016). In the next step, making professional judgments (MPJ), collected learning evidence is interpreted in relation to established standards (i.e., criterion-referenced assessment) or students’ progress made over time (i.e., pupil-referenced assessment). The final step, providing appropriate feedback, moves students forward through descriptive feedback that enables students to recognize their learning gap and monitor their own learning to close such gap. These four steps of CBA are interrelated with one another, rather than separated, and need to be implemented in a holistic way to fulfil the overriding aim of supporting student learning.

### Assessing YLLs

Assessing YLLs warrants a great deal of attention because of their unique age-related characteristics, which are generally categorized into three types: growth, literacy and vulnerability (McKay, 2006). First, YLLs are undergoing cognitive, social and emotional, as well as physical growth, which is nonlinear and dynamic (Berk, 2017). For example, they have short attention span, usually love physical activities, and are developing social awareness and a sense of self-esteem, which generates a strong demand for teachers to select or conduct appropriate assessment tasks (Patekar, 2021). Second, compared with older or adult learners, YLLs are still developing literacy skills in their first language when they are learning a foreign language, and this can have both conflicting and constructing influence on their literacy skills in the foreign language (Butler, 2016). Third, YLLs are particularly vulnerable to adults’ praise and criticism concerning their assessment performance. Their experience with assessments can have a long-lasting impact on their learning motivation, self-confidence, and learning outcome (Butler, 2019).

Against the backdrop of the above unique characteristics of YLLs, researchers have proposed principles for effective assessment of YLLs (Edelenbos and Vinjé, 2000; Hasselgreen, 2000; Cameron, 2001). Some key principles are summarized as follows: assessment tasks should fit with YLLs’ learning experience, reflecting those activities conducted in class; traditional achievement tests should not be viewed as the only form of assessment, instead, alternative forms of assessment, such as student portfolios, self-and peer assessment, need to be promoted for YLLs; assessment processes should help YLLs to monitor their language learning and develop their self-regulation abilities. CBA has been recognized as a practical solution to address such principles. It incorporates clear clarification of learning goals, multiple assessment methods, and quality feedback toward learning goals. Through CBA, YLLs can become aware of goals, develop positive learning attitudes, and gradually develop a sense of control over their own learning (Butler, 2021).

Given that assessment of YLLs is a newly emerging field (Hasselgreen, 2012), many areas have been underexplored, among which is CBA for YLLs, in particular. Limited research on this area has, however, highlighted that the implementation of CBA in young learner contexts is less than straightforward. For example, teachers of young learners fail to make assessment criteria explicit (Hild and Nikolov, 2011), frequently apply traditional assessment methods (e.g., objective tests; Prošić-Santovac et al., 2019; Yan et al., 2021), provide mostly evaluative feedback (e.g., marks) (Brumen et al., 2009), and mainly use assessment for summative purposes (Rixon, 2016). Moreover, research suggests that much can constrain teachers’ CBA enactment, such as teachers’ low assessment literacy (Vogt and Tsagari, 2014), lack of training in assessment (Patekar, 2021), limited opportunities for professional development (Lee et al., 2019), tight curriculum content (Mak and Lee, 2014), and an examination-oriented culture (Kaur, 2021). It could thus be said that although CBA is regarded as an effective approach to the improvement of YLLs’ learning, the implementation of CBA in local contexts is a challenging endeavor.

### L2 teachers’ beliefs and practices regarding CBA

Despite the aforementioned research efforts into the enactment of CBA in young learner contexts, teachers’ beliefs and practices about CBA for YLLs, as well as their relationship, remains largely under-investigated. Teacher beliefs, used interchangeably with teacher conceptions and teacher cognition, generally refer to “the unobservable cognitive dimension of teaching—what teachers know, believe and think” (Borg, 2003, p. 81). Teachers hold beliefs about various aspects of their work like teaching, learning, teachers, students, curriculum and materials (Borg, 2001), and their beliefs exist as a system wherein some core beliefs are stable and hard to change, while others are peripheral (Pajares, 1992). Studies have shown that teachers’ beliefs can exert a powerful influence on their instructional decisions (Burns et al., 2015; Li, 2020), though such beliefs may not always be reflected in their practices (Johnson, 1992; Dixon et al., 2011). Multiple factors, such as contextual complexities (e.g., class size, time constraints, authority’s influence), teachers’ teaching experience, and students’ needs, can determine the extent to which teachers can act according to their beliefs (Phipps and Borg, 2009; Roothooft, 2014). In this study, teachers’ CBA beliefs refer to teachers’ views toward the processes of CBA, including PA, collecting evidence, making professional judgments, and providing feedback, and teachers’ CBA practices are described as the enactment of these processes.

Research on L2 teachers’ beliefs and practices related to CBA has produced mixed results. There is evidence of a powerful effect that teachers’ beliefs have on the way they implement CBA practices (e.g., Mui So and Hoi Lee, 2011; Wang, 2017; Prošić-Santovac et al., 2019; Wu et al., 2021b). Mui So and Hoi Lee (2011), for instance, investigated English-as-a-second-language (ESL) secondary school teachers’ beliefs and practices of CBA for learning and found that their assessment practices consistently reflected their beliefs about the purpose of CBA. Similar congruence between teachers’ CBA beliefs and practices was identified in EFL contexts (Zhou and Deneen, 2016; Wu et al., 2021c). However, more studies have reported discrepancies teachers’ CBA beliefs and practices (e.g., Xu and Liu, 2009; Chen et al., 2014; Gan et al., 2018; Nasr et al., 2018; Vattøy, 2020; Wang et al., 2020; Mäkipää, 2021). For example, in their study of two university EFL teachers’ enactment of CBA, Chen et al. (2014) found that the teachers expressed positive attitudes toward students’ involvement in the assessment process, whereas in their actual practice, they seldom engaged students in self-and peer assessment. More recently, Vattøy (2020) interviewed ten secondary EFL teachers in Norway and found misalignment between teachers’ beliefs and practices regarding formative teacher feedback. These studies have also shown that L2 teachers’ CBA beliefs and practices are affected by individual factors (e.g., students’ needs, core beliefs held by teachers and their teaching experience) and sociocultural factors (e.g., policy support, class size, time constraints, prescribed curriculum and assessment culture).

One significant gap emerges from the existing studies on L2 teachers’ CBA beliefs and practices is that a majority of the previous studies were concerned mainly with secondary and university teachers, and less space has been devoted to teachers of YLLs. More research is needed regarding teachers’ beliefs and practices pertinent to CBA in young learner contexts. As discussed earlier, YLLs set themselves apart from older learners due to their special age-related characteristics. While previous research has offered insights into how secondary and university language teachers perceive and enact CBA for older learners, whether teachers of YLLs manifest similar beliefs and practices about CBA remains unknown. Given that teachers’ beliefs are context-dependent (Yu et al., 2020), further investigation into teachers’ beliefs and practices about CBA in young learner contexts is essential. Another important gap is that previous studies on L2 teachers’ CBA beliefs and practices merely focused on one or two aspects of CBA, with an intensive discussion on self-and peer assessment and teacher feedback (e.g., Chen et al., 2014; Vattøy, 2020; Mäkipää, 2021). To address this gap, two recent studies, conducted by Wang et al. (2020) and Wu et al. (2021c) respectively, have attempted to investigated language teachers’ beliefs about CBA in a more comprehensive way. Nonetheless, both studies were contextualized within university EFL classrooms. Thus, the current study, situated in the Chinese context, aims to investigate primary school EFL teachers’ beliefs and practices relating to the holistic process of CBA, including PA, collecting evidence, MPJ, and providing feedback.

## Research questions

Informed by the research gaps discussed above, this study seeks to address the following three questions:

RQ1: What beliefs do primary school EFL teachers hold about the processes of CBA?

RQ2: How do primary school EFL teachers implement CBA practices?

RQ3: To what extent do primary school EFL teachers’ beliefs about CBA align with their practices?

## Methodology

A questionnaire-based survey study was conducted to answer the research questions. Descriptions of the instrument, data collection, participants, and data analysis are provided in the ensuing sections.

### Instrument

Our research instrument, the Primary School English Teachers’ CBA questionnaire, was developed in a larger study on the implementation of CBA for young learners in China (Yan, 2020). The questionnaire comprised four sections: Perceived-purpose Scale, Perceived-process Scale, Practice Scale, and Demographic Information. The present study focused on the Perceived-process Scale and the Practice Scale, which examined teachers’ beliefs about the processes of CBA and their self-reported CBA practices, respectively.

Following Dörnyei and Taguchi (2010) guidelines, we first identified potential constructs of the Perceived-process Scale and the Practice Scale with reference to Davison and Leung (2009) framework of CBA. As discussed earlier, four dimensions are included in this framework: PA, collecting learning evidence, making professional judgments and providing appropriate feedback. The dimension collecting learning evidence was designed in this study to emphasize three types of assessment methods: SAO, planned assessment opportunities and FATs. Given the importance of distinguishing descriptive feedback from evaluative feedback (Wiliam, 2010), both types of feedback were included in the dimension providing appropriate feedback. Thus, seven potential constructs were included in both the Perceived-process Scale and the Practice Scale: PA, using SAO, using planned assessment opportunities, using FATs, MPJ, providing descriptive feedback (PDF), and providing evaluative feedback (PEF).

Forty-four items were initially generated for both scales, most of which were drawn from the Beliefs about Assessment and Evaluation questionnaire (Rogers et al., 2007), the Classroom Assessment questionnaire (Cheng et al., 2004), the “Learning How to Learn” project’s questionnaire (James and Pedder, 2006), and the Assessment Practices Inventory (Zhang and Burry-Stock, 2003). Some items were generated on the basis of the semi-structured interviews conducted with three primary school EFL teachers who did not participate in the main study. The first researcher interviewed the three teachers individually, during which an open, comprehensive topic was discussed: What do you think about the purposes of teachers’ classroom assessment and what assessment practices do you employ when assessing your students? The teachers’ responses were used as a source for the item pool. For example, when asking about assessment practices, one teacher described that “I frequently check whether students have mastered what they learned in class through classroom tests, dictation and recitation.” This guided the writing-up of the questionnaire items such as “Teachers collect evidence of learning through classroom tests” and “Teachers collect evidence of learning through dictation.” Two 6-point Likert scales were established to study teachers’ beliefs about CBA in the Perceived-process Scale (1 = Not important at all, 2 = Not important, 3 = Somewhat important, 4 = Important, 5 = Very important, 6 = Completely important), and their self-reported CBA practices in the Practice Scale (1 = Never, 2 = Very rarely, 3 = Rarely, 4 = Occasionally, 5 = Frequently, 6 = Always), respectively.

Finally, to examine the content validity, two experts and a group of postgraduates were invited to provide feedback on the content relevance, clarity and comprehensiveness of the two scales, based on which two items were dropped, retaining 42 items, and some items were revised. The items were originally generated in English, and then translated from English to Chinese by the first author and a doctoral student, using a back-translation method (Nunan and Bailey, 2009). The questionnaire was then piloted with 26 primary school English teachers, who did not participate in the main study and provided comments on questionnaire clarity and administration procedures. Modifications were made and a finalized questionnaire was obtained (Appendix A).

### Data collection

The first author collected the questionnaire data. A convenience sampling strategy that is commonly used in L2 research (Dörnyei, 2007) was used to collect questionnaire responses from teachers who were teaching EFL at primary schools in China. Specifically, the target participants were primary school EFL teachers from one municipality and one province in China, to whom the first researcher had access. To approach these participants, an information sheet was first sent to six primary school EFL teaching advisors in the two places, who had the responsibility of providing guidance to teachers’ daily teaching and were in charge of all teachers in their own districts. An online questionnaire invitation was then sent out to primary school teachers through the advisors. A total number of 312 questionnaires were received. Of those, 117 questionnaires with an obvious response set (i.e., almost the same answers for all the items) were excluded, leaving 195 valid responses.

### Participants

As Table 1 shows, a majority of the teacher participants were female (96.9%, *n* = 189), and most teachers were aged under 40 years (72.9%, *n* = 142). Over three quarters of the teachers held a bachelor’s degree (78.5%, *n =* 153), with a small minority holding a master’s degree and a degree lower than the Bachelor (21.5%, *n* = 42). About half of the participants had 6 to 20 years’ experience of teaching EFL to primary school students (52.3%, *n* = 102); 33.8% (*n* = 66) had less than 5 years and 13.5% (*n* = 27) had over than 20 years. The number of teachers from public schools (88.2%, *n* = 172) was larger than that from private schools (11.8%, *n* = 23). Of the participants, 69.2% (*n* = 136) taught lower grade levels (Grades 1, 2, 3 and 4), and a similar number taught higher grade levels (Grades 5 and 6) (64.1%, *n* = 125). Most participants had completed a course on assessment or received training in assessment (64.6%, *n* = 126).

**Table 1:** Demographic information of the teacher participants.

Demographics	Groups	Number	Percentage
Gender	Male	6	3.1
	Female	189	96.9
Age	Under 30	51	26.2
	31–40	91	46.7
	41–50	45	23.1
	Over 50	8	4.0
Educational qualification	Under Bachelor	30	15.4
	Bachelor	153	78.5
	Master	12	6.1
Teaching experience	Less than 1 year	18	9.2
	1–5 years	48	24.6
	6–10 years	22	11.3
	11–15 years	45	23.1
	16–20 years	35	17.9
	Over 20 years	27	13.8
School type	Public	172	88.2
	Private	23	11.8
Grade level	Grade1	14	6.7
	Grade 2	8	4.1
	Grade 3	49	25.1
	Grade 4	65	33.3
	Grade 5	67	34.4
	Grade 6	58	29.7
Assessment-related course or training	No	69	35.4
	Yes	126	64.6

### Data analysis

Questionnaire data was analyzed using SPSS 24. Missing data, outliers, and normality distribution were checked first. Exploratory factor analysis (EFA) was then conducted to explore the underlying constructs of the Perceived-process Scale and the Practice Scale. Assumptions were checked through Kaiser-Meyer-Olkin’s (KMO) Test of Sampling Adequacy and Bartlett’s Test of Sphericity. Factors were extracted using principal axis factoring with non-orthogonal rotation (Direct Oblimin, *δ* = 0) solution. A factor loading with a minimum absolute value of 0.30 was required. The results indicated a 37-item seven-factor solution and a 31-item six-factor solution for the Perceived-process Scale and the Practice Scale, respectively, (underlying constructs of the perceived-process scale and practice scale).

To answer RQ1 regarding teachers’ beliefs about CBA, descriptive analysis was conducted to obtain a general understanding of teachers’ beliefs about the seven factors. And repeated-measures ANOVA was calculated to examine the differences of teachers’ beliefs among different factors. The same analyzes were run to answer RQ2 regarding teachers’ self-reported CBA practices. To answer RQ3 regarding teachers’ belief-practice relationship, correlation analysis and multiple regression analysis were carried out. Correlation analysis was performed to examine whether there existed a direct relationship, and the latter was performed to explore how well teachers’ CBA beliefs could predict their practices.

## Results

### Underlying constructs of the perceived-process scale and practice scale

#### Underlying constructs of the perceived-process scale

In the process of EFA of 42 items on the Perceived-process Scale, five items yielded cross-loadings over 0.30 on more than one factor and thus were discarded. The cross-loading of four items (Item 1.12, Item 1.26, Item 1.28 and Item 1.31) may be explained by the fact that the four key steps of CBA are interrelated with one another. For instance, Item 1.12 ‘Teachers collect evidence of learning through classroom observation’, which was initially designed as a potential item for the construct using SAO, cross-loaded onto the construct PDF. The potential construct using SAO emphasizes that incidental assessment opportunities like teacher observation can be generated by teachers to timely obtain learning evidence and provide immediate feedback (Turner and Purpura, 2016). From a conceptual perspective, this construct not only addresses the method of evidence collection but also the formative purpose of using such method, and thus is related to the potential construct PDF that has a focus on evidence use. Therefore, it can be deduced that the cross-loading of Item 1.12 was justified. Besides, the cross-loading of another item, Item 1.7, may be associated with construct clarity.

According to Davison and Leung (2009), selecting appropriate assessments is an important element of the construct PA, based on which Item 1.7 was generated as “Teachers select appropriate assessment methods according to students’ needs when PA.” This potential item, however, cross-loaded onto the construct PA and the construct using SAO. This finding indicates that the construct PA appears to require clarification and offers a possibility of considering selecting appropriate assessment methods as an element of collecting learning evidence. After the five cross-loading items were removed, seven factors were extracted, with a cumulative contribution of 72.54% (KMO = 0.934, *df* = 666, *p* < 0.001). Cronbach’s alpha coefficients (*α*) of the seven factors ranged from 0.834 to 0.953, indicating good internal consistency.

Table 2 shows that Factor 1, PDF contained seven items (*a* = 0.953) regarding using feedback to improve student learning, such as identifying strengths and weaknesses in relation to learning goals and finding solutions to help students improve their learning. Factor 2 included six items (*a* = 0.911), revealing how regular instructional activities like oral presentations and role plays can be conducted as assessment opportunities, where students often play an active role in the assessment process, for instance, being involved in self-and peer assessment. These six items were originally designed to be covered by the construct planned assessment opportunities, which addresses that teachers can design or plan instruction-embedded activities to elicit student learning. However, in order to highlight students’ active role, this factor was relabeled student-involving assessment opportunities (SIA). Factor 3, using FAT, included five items (*a* = 0.886) showing the use of more formal types of assessments (e.g., classroom tests, dictation, oral reading and reciting). Factor 4, PEF, included four items (*a* = 0.834) focusing on providing feedback to students and parents about their achievement through scores and grades. Factor 5, PA, covered six items (*a* = 0.847) in relation to establishing and sharing instructional objectives and assessment criteria with students. Factor 6, MPJ, contained three items (*a* = 0.889) showing how judgments of students’ performance could be made by comparing their performance to pre-set learning goals or their previous performance. Factor 7, using SAO, included six items (*a* = 0.881) revealing how informal and unplanned assessments (e.g., teacher questioning, teacher-student conversations) could be embedded in daily instruction to modify teaching and provide immediate feedback to students. It can be seen that the original seven constructs of the Perceived-process Scale were retained with EFA.

**Table 2:** Results of the perceived-process scale.

Items	Factor loading						
	Factor 1	Factor 2	Factor 3	Factor 4	Factor 5	Factor 6	Factor 7
	PDF	SIA	FAT	PEF	PA	MPJ	SAO
Item 1.42	0.890						
Item 1.41	0.880						
Item 1.40	0.854						
Item 1.39	0.828						
Item 1.38	0.742						
Item 1.37	0.696						
Item 1.27	0.318						
Item 1.19		0.807					
Item 1.18		0.799					
Item 1.17		0.653					
Item 1.20		0.638					
Item 1.16		0.546					
Item 1.15		0.540					
Item 1.22			0.809				
Item 1.23			0.766				
Item 1.24			0.718				
Item 1.21			0.641				
Item 1.25			0.352				
Item 1.34				0.754			
Item 1.33				0.602			
Item 1.35				0.568			
Item 1.36				0.394			
Item 1.1					0.788		
Item 1.2					0.721		
Item 1.5					0.519		
Item 1.3					0.427		
Item 1.4					0.394		
Item 1.6					0.305		
Item 1.30						0.809	
Item 1.29						0.712	
Item 1.32						0.603	
Item 1.11							−0.707
Item 1.10							−0.694
Item 1.8							−0.647
Item 1.9							−0.592
Item 1.14							−0.435
Item 1.13							−0.371

#### Underlying constructs of the practice scale

In the process of EFA of the 42 items on the Practice Scale, four items had lower loadings of 0.30, four items loaded on more than one factor, and another three items were identified as outlying ones at they were unrelated to other items on the same factor. The cross-loading of the items on the Practice Scale (Item 2.10, Item 2.11, Item 2.25, Item 2.28) is likely to be explained by the precision of language wording. For example, Item 2.25 ‘I take account of students’ language knowledge (e.g., vocabulary, grammar) when interpreting assessment data’, as a potential item for the construct making professional judgments, was cross-loading onto the construct using FAT. A careful examination of this item showed qualitative difference in wording as compared to some other potential items for the same construct. The use of “take account of” seemed to indicate that this item focused on what to be assessed. However, other items like Item 2.29, and Item 2.30 used “compared students’ current performance against” to suggest a clear focus on how to make sense of assessment data. The wording difference, as a result, might affect participants’ interpretation of the items. In the final solution, 11 items were discarded and six factors were extracted, explaining 71.48% of variance (KMO = 0.925, *df* = 465, *p* < 0.001). Cronbach’s Alpha coefficients (*a*) of the six factors ranged from 0.848 to 0.954.

As Table 3 shows, Factor 1, PDF, contained six items (*a* = 0.954) reflecting teachers’ use of detailed feedback to help students recognize their strengths and weaknesses in learning and move their learning forward. Factor 2, PA, with seven items (*a* = 0.885), reflected teachers’ practices of establishing and sharing instructional objectives and assessment criteria with students as well as selecting appropriate assessment methods. Factor 3, SIA, with five items (*a* = 0.871), reflected teachers’ use of instruction-embedded assessment opportunities where students played a major role (e.g., self-assessment, peer assessment). Factor 4, FAT, with four items (*a* = 0.848), showed teachers’ use of FATs to collect learning evidence (e.g., classroom tests, dictation). Factor 5, SAO, with five items (*a* = 0.859), revealed teachers’ use of incidental instruction-embedded assessments (e.g., observation, oral questioning). Factor 6, MPJ, with four items (*a* = 0.879), showed how teachers made professional interpretation of assessment information. The results show that only six constructs were retained with EFL, with the original construct PDF being dismissed. The reason is that two initial items (Item 3.35 and Item 3.36) of PDF had a lower loading of 0.30 during the process of factoring. Another two items (Item 3.34 and Item 3.34) of PDF loaded onto the factor FAT and were regarded as outlying variables because these two items focused on how teachers reported students’ performance while the four items of the factor FAT were related to teachers’ use of formal tasks to evaluate students’ performance. This was confirmed by the reliability coefficient for the factor FAT, showing that Cronbach’s alpha became higher if Item 3.33 and Item 3.34 were deleted.

**Table 3:** Results of EFA of the practice scale.

Items	Factor loading					
	Factor 1	Factor 2	Factor 3	Factor 4	Factor 5	Factor 6
	PDF	PA	SIA	FAT	SAO	MPJ
Item 2.41	0.952					
Item 2.38	0.865					
Item 2.42	0.845					
Item 2.40	0.803					
Item 2.39	0.754					
Item 2.37	0.722					
Item 2.5		−0.847				
Item 2.1		−0.817				
Item 2.2		−0.772				
Item 2.7		−0.706				
Item 2.6		−0.687				
Item 2.3		−0.686				
Item 2.4		−0.520				
Item 2.16			0.865			
Item 2.17			0.839			
Item 2.15			0.721			
Item 2.18			0.690			
Item 2.14			0.436			
Item 2.22				0.826		
Item 2.21				0.662		
Item 2.23				0.639		
Item 2.24				0.637		
Item 2.13					−0.557	
Item 2.19					−0.554	
Item 2.12					−0.501	
Item 2.20					−0.477	
Item 2.9					−0.353	
Item 2.30						−0.702
Item 2.32						−0.673
Item 2.29						−0.500
Item 2.31						−0.477

### Teachers’ CBA beliefs

Descriptive data of teachers’ beliefs of the CBA processes are displayed in Table 4. The seven factors derived from the Perceived-process Scale all had a mean score higher than 4.0 (“Important”), indicating that the teachers believed that the various processes of CBA were important for the enhancement of student learning. Specifically, they agreed that PA and PDF were especially valuable, judging from the mean scores that exceeded 5.0 (“Very important”).

**Table 4:** Descriptive data of teachers’ CBA beliefs.

CBA beliefs	*M*	*SD*
Beliefs of planning assessment (PA)	5.07	0.55
Beliefs of using spontaneous assessment opportunities (SAO)	4.83	0.57
Beliefs of using student-involving assessment opportunities (SIA)	4.67	0.62
Beliefs of using formal assessment tasks (FAT)	4.86	0.56
Beliefs of making professional judgments (MPJ)	4.96	0.56
Beliefs of providing descriptive feedback (PDF)	5.08	0.56
Beliefs of providing evaluative feedback (PEF)	4.64	0.60

One-way repeated-measures ANOVA was conducted to examine differences among teachers’ beliefs regarding the seven factors. The assumption of normal distribution was satisfied given that the values of skewness (from −0.421 to 0.209) and kurtosis (from −0.251 to 1.037) fell within the cut-off values of|3.0|and|8.0|respectively (Kline, 2011). Mauchly’s Test showed that the assumption of sphericity had been violated, *x*^2^ (20) = 68.888, *p* < 0.001, so Greenhouse–Geisser of Huynh-Feldt (*ε* = 0.909) was applied to adjust degrees of freedom. The results of ANOVA showed that there were significant differences in the importance attached to the CBA processes, *F* (5.289, 1026.070) = 42.694, *p* < 0.001, *η*^2^ = 0.18. Follow-up pairwise comparisons with Bonferroni corrections indicated that all pairwise differences were significant (*p* < 0.05) excepted four paired comparisons, namely PA and PDF (*p* > 0.05), SAO and FAT (*p* > 0.05), SIA and PEF (*p* > 0.05), FAT and MPJ (*p* > 0.05). This suggested that the teachers considered PA and PDF to be the most important CBA processes in promoting student learning. As for the specific assessment methods for learning evidence collection, they placed greater emphasis on SAO (e.g., teacher questioning, observations) and FAT (e.g., classroom tests, textbook exercises, recitation), whereas a relatively lower preference was shown for SIA (e.g., self-assessment and peer assessment). They also believed strongly in the value of MPJ (e.g., comparing learning evidence against pre-set goals). Comparatively, from teachers’ perspective, SIA and PEF were the least important CBA processes.

### Teachers’ CBA practices

Table 5 presents the descriptive data of teachers’ self-reported CBA practices. The mean scores for PA, SAO, MPJ and PDF exceeded 5.0 (“Frequently”) and those for SIA and FAT exceeded 4.0 (“Occasionally”). The results revealed that, on average, the teachers reported frequent-use of PA, SAO, MPJ and PDF and occasional-use of SIA and FAT.

**Table 5:** Descriptive data of teachers’ CBA practices.

CBA practices	*M*	*SD*
Practices of planning assessment (PA)	5.23	0.55
Practices of using spontaneous assessment opportunities (SAO)	5.07	0.53
Practices of using student-involving assessment opportunities (SIA)	4.55	0.65
Practices of using formal assessment tasks (FAT)	4.96	0.60
Practices of making professional judgments (MPJ)	5.04	0.53
Practices of providing descriptive feedback (PDF)	5.04	0.56

One-way repeated measures ANOVA was applied to examine the differences among the six factors. The data met the assumption of normal distribution (the values of skewness ranging from −0.285 to 0.150, and the values of kurtosis from −0.704 to 0.467), but the assumption of sphericity was violated, *x*^2^ (14) = 79.825, *p* < 0.001.Therefore, Greenhouse–Geisser of Huynh-Feldt (*ε* = 0.877) was applied. Significant differences were identified in the frequency of different CBA practices, *F* (4.277, 829.715) = 66.582, *p* < 0.001, *η*^2^ = 0.256. Follow-up pairwise comparisons with Bonferroni corrections showed that all pairwise differences were significant (*p* < 0.05) except six paired comparisons, namely, SAO and FAT (*p* > 0.05), SAO and MPJ (*p* > 0.05), SAO and PDF (*p* > 0.05), FAT and MPJ (*p* > 0.05), FAT and PDF (*p* > 0.05), MPJ and PDF (*p* > 0.05). The results revealed that, according to the teachers’ self-report, PA was the most frequently used CBA practice. As for collecting learning evidence, the teachers frequently used assessment methods of SAO (e.g., oral questioning and observations) and FAT (e.g., classroom tests and recitation tasks), while SIA (e.g., self-assessment and peer assessment) was less frequently used. Meanwhile, they reported frequent use of MPJ (e.g., made judgments of students’ performance against learning objectives or their previous performance) and PDF (e.g., providing feedback to students to identify learning strengths and weaknesses). In general, SIA was the least frequently used CBA practice by the teachers.

In summary, Chinese primary school EFL teachers in this study showed strong positive attitudes toward CBA, perceiving that PA and PDF were the most important assessment processes. While they emphasized the value of using multiple assessment methods, they placed more importance on SAO and FATs than student-involving assessment opportunities. MPJ were also considered as imperative for improving student learning. PEF, as opposed to PDF, gained the least popularity among teachers. A similar pattern was identified in their self-reported CBA practices, as they reported the most frequent practice of PA, multiple use of assessment methods to collect students’ learning evidence (with a heavy reliance on SAO and FAT), as well as frequent practice of MPJ and PDF. Such a similar pattern seemed to indicate alignment between teachers’ beliefs about CBA and their assessment practices.

### Relationship between teachers’ CBA beliefs and practices

To examine the relationship between teachers’ beliefs and their self-reported practices related to CBA, correlation analysis was first conducted. As seen in Appendix B, each of the seven factors of teachers’ beliefs about CBA was positively and significantly correlated with the six factors of teachers’ self-reported CBA practices. Thus, six multiple regression analyzes using each of the six self-reported CBA practices as dependent variables were conducted, with the seven factors of teachers’ beliefs about CBA being set as predictors (see Table 6). The seven factors of teachers’ beliefs were found to have a significant effect on teachers’ CBA practices.

**Table 6:** Predictions of teachers’ CBA beliefs on CBA practices.

Predictors	Teachers’ CBA practices					
	Practice-PA	Practice-SAO	Practice-SIA	Practice-FAT	Practice-MPJ	Practice-PEF
Beta (*β*)						
*Teachers’ CBA beliefs*						
Belief-PA	0.369***	0.095	−0.141	−0.012	0.088	−0.065
Belief-SAO	−0.048	0.066	0.036	−0.003	−0.050	−0.077
Belief-SIA	0.086	0.157	0.437***	−0.024	0.045	0.199
Belief-FAT	0.028	0.092	0.014	0.417***	0.076	0.077
Belief-MPJ	0.005	−0.011	0.062	0.108	0.253**	0.052
Belief-PDF	0.273**	0.349***	−0.090	−0.008	0.201*	0.491***
Belief-PEF	−0.120	−0.087	0.109	0.047	0.026	−0.004
*R* ^2^	0.311	0.331	0.193	0.235	0.299	0.376
Adjusted *R*^2^	0.286	0.306	0.163	0.206	0.273	0.352
*df*	(7,187)	(7,187)	(7,187)	(7,187)	(7,187)	(7,187)
*F*	12.078***	13.225***	6.398***	9.191***	11.410***	26.071***

***p < 0.001, **p < 0.01, *p < 0.05.

First, the results of multiple regression showed a significant model for the practice of PA, *R*^2^ = 0.311, adjusted *R*^2^ = 0.286, *df* = (7,187), *F* = 12.078, *p* < 0.001. The PA practice was predicted by the factors regarding teachers’ beliefs about PA (*β* = 0.369, *p* < 0.001) as well as PDF (*β* = 0.273, *p* < 0.01). Second, the results showed a significant model for the practice of SAO, *R*^2^ = 0.331, adjusted *R*^2^ = 0.306, *df* = (7,187), *F* = 13.225, *p* < 0.001. Such practice was predicted by teachers’ beliefs about SAO (*β* = 0.349, *p* < 0.001). Third, a significant model was identified for the practice of SIA, *R^2^* = 0.232, adjusted *R*^2^ = 0.193, *df* = (7,187), *F* = 6.398, *p* < 0.001. Likewise, this practice was predicted by the factor of teachers’ beliefs about SIA. Fourth, a significant model was found for the practice of FAT, *R*^2^ = 0.235, adjusted *R*^2^ = 0.206, *df* = (7,187), *F* = 9.191, *p* < 0.001. In the same vein, the practice was predicted by teachers’ beliefs about FAT (*β* = 0.417, *p* < 0.001). Regarding the practice of MPJ, a significant model was also identified, *R*^2^ = 0.299, adjusted *R*^2^ = 0.273, *df* = (7,187), *F* = 11.410, *p* < 0.001). Such practice was predicted by teachers’ beliefs of MPJ (*β* = 0.253, *p* < 0.01) and PDF (*β* = 0.201, *p* < 0.05). Finally, a significant model was identified for the practice of PDF, *R*^2^ = 0.376, adjusted *R*^2^ = 0.352, *df* = (7,187), *F* = 23.044, *p* < 0.001), showing that the factor of teachers’ beliefs of PDF was a significant predictor (*β* = 0.491, *p* < 0.001).

Overall, the above results revealed strong relationships between Chinese primary school EFL teachers’ beliefs about CBA and their assessment practices. Their beliefs regarding PA, SAO, SAI, FAT, MPJ and PDF were found to be important predictors of the related assessment practices.

## Discussion

Results of this study indicated that Chinese primary school EFL teachers generally held positive attitudes toward the various processes of CBA, and also reported that they attempted to enact CBA practices in their classrooms to support learning of YLLs. Their beliefs about CBA seemed to have a powerful influence on their assessment practices. Overall, this study contributes to the literature by comprehensively examining teachers’ self-reported beliefs and practices regarding CBA for young EFL learners in the Chinese context. Each research question is addressed in light of the findings.

Regarding the first research question, the findings revealed that Chinese primary school EFL teachers agreed on the importance of various processes of CBA (e.g., PA, collecting learning evidence, MPJ, providing appropriate feedback). Similar findings have been reported in the literature (e.g., Wang et al., 2020; Golzar et al., 2022). For example, in the recent study by Golzar et al. (2022), Afghan university EFL teachers perceived that student-involving assessment methods (e.g., self-and peer assessment) and quality feedback were of great value in student learning improvement. However, Indonesian EFL teachers in Puad and Ashton (2021) study mainly viewed CBA from a summative perspective, showing negative attitudes about self-and peer assessment and believing in the value of scores and grades in making students accountable. This differs from the finding of the current study. It seems that the teaching and learning context is an important factor on teachers’ beliefs about CBA.

As for the specific processes of CBA, the teachers in this study placed the greatest importance on PA, addressing the value of establishing and sharing clear instructional objectives and success criteria with students. This finding is close to Wu et al. (2021b) investigation in the Chinese university EFL context, where the teachers attached importance to communicating learning goals and success criteria to students. The significance of articulation of learning goals and success criteria, as suggested by previous research (Timperley and Parr, 2009; Balloo et al., 2018), is that it enables students to be truly engaged in the process of deep learning rather than surface-level learning that has a focus on task completion, which is important for students’ self-regulatory capacity. The teachers in this study also believed that, when making judgments, it was important to compare students’ learning performance against pre-set learning objectives or students’ previous learning progress. Such beliefs appear to be held by researchers like Airasian and Abrams (2003) and Jacobs and Renandya (2019), who highly emphasize the value of criterion-referenced assessment and pupil-referenced assessments to mitigate the undesirable negative impact of competition, diagnose students’ learning needs, and to identify strategies for learning improvement. In addition, the teachers in this study placed greatest emphasis on the value of descriptive feedback to help students understand what was necessary for achievement and how to overcome difficulties in learning. In comparison, they did not place a high value on evaluative feedback. The findings here are similar to Brumen and Cagran (2011) research where the teachers from three European countries (Czech Republic, Slovenia and Croatia) believed that YLLs should be provided with more descriptive and individual feedback rather than numerical grades. As Brumen and Cagran (2011) suggested, young learners would benefit more directly from descriptive feedback on their learning progress and language development.

However, when it comes teachers’ beliefs about collecting learning evidence, complex findings were identified in this study. The teachers placed considerable value on the use of multiple assessment methods, including SAO embedded in daily instruction (e.g., teacher questioning), FATs (e.g., classroom tests), and student-involving assessment opportunities (e.g., self-and peer assessment). This finding has also been seen in other studies (Shohamy et al., 2008; Troudi et al., 2009). Multiple assessment methods have the potential to respond to a wide range of L2 students’ learning needs (Leung, 2005). In primary schools in China, teachers usually teach large-size classes with up to 50 students (Wang, 2009; Wu et al., 2021b), leading them to use different forms of assessment to address students’ learning needs. Despite their positive attitudes toward multiple assessment methods, the teachers in this study perceived student-involving assessments as less important as compared to other assessment types. The relative conservative beliefs about student-involving assessments like self-and peer assessment might reflect the teachers’ concern over the subjectivity and validity of these assessment methods. Indeed, although some empirical evidence has indicated the effectiveness of such assessment methods in promoting YLLs’ autonomy (e.g., Butler and Lee, 2010; Liu and Brantmeier, 2019), there is widespread beliefs among teachers that YLLs are too immature for evaluating and self-regulating their own learning, as Butler (2019) noted. Similar traditional beliefs about student-involving assessments have been reported in previous empirical studies with YLLs (Tsagari, 2016). Actually, even for older learners, L2 teachers tend to believe that learners do not seem to have sufficient knowledge to accurately assess their own learning (Puad and Ashton, 2021).

Regarding the second research question, the findings of the present study are generally different from those found in Rixon (2016) international survey study where the teachers from over 100 countries across the globe were investigated regarding their assessment practices in young learner classrooms. In Rixon’s study, the teachers mainly used assessment for summative purposes, failing to make full use of assessment information to support YLLs’ English language learning. By contrast, in the present study, Chinese primary school EFL teachers attempted to enact various CBA practices to enhance YLLs’ language learning, such as PA, using multiple assessment methods, and PDF. Such findings are closer to Lee et al. (2019) study that showed the primary school English teachers’ attempts to implement CBA in writing classrooms to benefit student learning in the Hongkong context.

The findings about Chinese primary school EFL teachers’ attempts to implement CBA practices, despite being self-reported, are also encouraging for Chinese educational policy makers, which suggest that teachers have tried to translate into action principles of CBA for learning. In China, CBA has begun to attract language assessment experts and researchers’ attention since the beginning of the new century, and has become a policy-support practice in the Chinese educational system almost at the same time (Gu, 2012). At the primary school level, CBA, as an assessment initiative, was incorporated into the English Curriculum Standards for Compulsory Education (ECSCE) (2011 version; MOE, 2011), aimed at promoting learner autonomy through the integration of CBA into regular instruction. Such an initiative has been readdressed in the newly published ECSCE (2022 version; MOE, 2022). In this study, a series of CBA practices (e.g., clarifying learning goals and success criteria, using multiple assessment methods, MPJ and PDF) had been implemented in young learner classrooms, as reported by the teachers. These findings revealed that Chinese primary school EFL teachers had provided CBA opportunities for young learners to improve their learning. But it has to be borne in mind that all this was based on what they reported.

Nevertheless, this study found that, among the various CBA practices, the teachers reported the lowest frequency of adopting student-involving assessment opportunities like self-and peer assessments; by contrast, teacher-centered FATs were more frequently conducted with YLLs. This is in accordance with research that has identified that student-involving assessment opportunities do not have much of a presence in practice in L2 classrooms (Saito and Inoi, 2017). Based on teachers’ self-reports, it appeared that Chinese primary school EFL teachers did not provide genuine opportunities for young learners to take responsibility for their own learning. This may in part be due to the traditional Chinese culture of teaching and learning, where teachers are conceptualized as the authoritative figure, and students are regarded as passive recipients of knowledge (Carless, 2011). A Chinese saying, ‘being a teacher for only 1 day entitles one to lifelong respect from the student that befits his/her father’ (yiri weishi zhongshen weifu), expresses this hierarchical teacher-student relationship. Having been influenced by this culture throughout their own student life, Chinese EFL teachers are likely to develop firm beliefs regarding the teacher and student roles (Cheng et al., 2021; Sun and Zhang, 2021; Zhang and Sun, 2022). As such, when conducting assessment, teachers tend to still take a dominant role in assessment and monitoring students’ learning despite their beliefs about the beneficial impact of student-involving assessments.

The less frequent use of student-involving assessments might also be attributed to teachers’ lack of CBA literacy. Although the majority of teachers in this study reported that they had received assessment-related training or attended related courses, it seems possible that insufficient CBA content had been provided. As Xu and Brown (2017) study showed, Chinese university EFL teachers had insufficient CBA training in both pre-service and in-service courses. Hence the lack of CBA training might hinder teachers from developing essential assessment literacy to translate CBA principles into practice. Previous literature has indicated that CBA-literature teachers will be committed to embedding student-involving assessment opportunities into instruction in an ongoing manner (Dixon et al., 2020).

Regarding the third research question, the findings from the descriptive analyzes revealed that teachers’ stated beliefs and practices regarding CBA were generally aligned in certain aspects. For example, the teachers highly valued the clarification of learning objectives and success criteria, professional judgments of student learning and the provision of descriptive feedback, which were reflected in their frequent practices of these CBA processes. Similar finding has been reported by Wang et al. (2020) study, showing that the teachers’ practices of making learning explicit matched their beliefs about creating a supporting learning environment and clarifying success criteria. In addition, in the present study, the teachers’ beliefs on the use of different assessment methods were largely consistent with their practices. The teachers placed the least value on student-involving assessment opportunities, which was correspondingly reflected in their least frequent practice of SIA. This finding is similar to Wu et al. (2021b) study, where the teachers’ beliefs and practices were aligned regarding empowering students in the assessment process. Furthermore, the findings of multiple regression analyzes demonstrated that Chinese primary school EFL teachers’ beliefs about CBA were important predictors of their assessment practices, echoing the powerful influence of teachers’ beliefs on their instructional behaviors (Borg, 2019). The finding of this study is generally like that of Brown et al. (2015) study, which reports alignment between teachers’ assessment beliefs and their self-reported assessment practices in the Indian context. Nonetheless, it needs to be kept in mind that there is no evidence in this study as to teachers’ actual classroom assessment practices. It could be that, in actual classroom settings, teachers have not fully implemented CBA practices as what they have reported in the survey, because teachers’ self-reported teaching practices do not necessarily reflect their actual classroom behavior (Chen et al., 2012). Further research is needed to explore the beliefs/practice nexus.

## Conclusion

Chinese primary school EFL teachers were found to place considerable value on the various processes of CBA for YLLs and reported that they had made attempts to implement CBA practices to facilitate the learning of YLLs. However, it would seem that their CBA practices need to be expanded to incorporate student-involving assessment opportunities, which was also reflected in their beliefs that such assessment opportunities were less important than FATs. In general, the teachers’ stated CBA beliefs and practices were in alignment.

Three pedagogical implications are drawn. First, it is recommended that teachers design student-involving assessment opportunities contextualized to the learning objectives of a particular lesson or unit. To maximize the benefits of building young learner autonomy, it is advisable for teachers to guide students to understand the learning objectives and success criteria using learner-friendly languages, and to cooperate with students to reflect on and monitor language knowledge and skills that they have mastered. Second, given the powerful impact of teachers’ beliefs on their CBA practices, it is suggested that teacher educators provide student teachers with sufficient experiences with CBA, and school administrators establish professional learning communities where meetings are organized to convey CBA principles and teachers can share their assessment experiences. This helps teachers to form positive beliefs about CBA, which, in turn, could motivate them to well utilize CBA as a pedagogical practice. Third, quality pre-service and in-service professional training programs are needed to help teachers master CBA-related knowledge and skills, especially those related to student-involving assessment opportunities. In this way, teachers will become CBA literate and can implement CBA practices more effectively.

Future studies that collect data from multiple sources, such as classroom observations, teacher interviews and teaching documents, are needed to validate teachers’ self-reported CBA practices. tMore research is needed to examine students’ beliefs about CBA and the consistency between students’ and teachers’ perspectives. Research on teachers’ experience with CBA-related training and the effect on their CBA practices is also warranted.

## Data availability statement

The original contributions presented in the study are included in the article/Supplementary material, further inquiries can be directed to the corresponding author.

## Ethics statement

The studies involving human participants were reviewed and approved by The University of Auckland Human Ethics Committee. Written informed consent to participate in this study was provided by the participants' legal guardian/next of kin.

## Author contributions

QY: conceived of the initial idea, designed the study, collected and analyzed the data, and drafted of the manuscript. LZ and HD: revised and proofread the manuscript. All authors agreed to the final version before LZ got it ready for submission as the corresponding author.

## Funding

This study is funded by a research grant from the Chongqing Social Sciences Planning Office for the Foreign Language Project (grant no. 2021WYZX43) and a research grant from the Fundamental Research Funds for the Central Universities in China (grant no. 2022CDJSKJC03).

## Conflict of interest

The authors declare that the research was conducted in the absence of any commercial or financial relationships that could be construed as a potential conflict of interest.

## Publisher’s note

All claims expressed in this article are solely those of the authors and do not necessarily represent those of their affiliated organizations, or those of the publisher, the editors and the reviewers. Any product that may be evaluated in this article, or claim that may be made by its manufacturer, is not guaranteed or endorsed by the publisher.

## Supplementary material

The Supplementary material for this article can be found online at: https://www.frontiersin.org/articles/10.3389/fpsyg.2022.1051728/full#supplementary-material
