# Supplementary material for: Exploring classroom-based assessment for young EFL learners in the Chinese context: Teachers’ beliefs and practices
Source: Front Psychol. 2022 Nov 14;13:1051728. doi: 10.3389/fpsyg.2022.1051728 (PMC9702076; doi:10.3389/fpsyg.2022.1051728)
Supplement: Supplementary file 1 [file Data_Sheet_1.docx]

**Appendixes**

**Appendix A. The Primary School English Teachers’ CBA Questionnaire**

Dear teachers,

This questionnaire is to gain an understanding of Chinese EFL teachers’ conceptions and practices regarding classroom assessment of young EFL learners. Please respond to each item by filling the blanks or ticking the appropriate options. Please *choose only one answer for all the multiple-choice questions except for those particularly labelled*. There is no right or wrong answers. *All the information you provide will be treated in confidence and used for research purposes only*. Thanks very much for your support.

**Your conceptions of classroom-based assessment processes**

The following statements address the processes that teachers use when assessing their students in classrooms. Please indicate your opinion by using the following rating scale:

1=Not important at all 2=Not important 3=Somewhat important 4=Important 5=Very important 6**=** Completely important

| **1.1** | Teachers should identify instructional objectives when designing language activities. | 1 | 2 | 3 | 4 | 5 | 6 |
| --- | --- | --- | --- | --- | --- | --- | --- |
| **1.2** | Teachers establish instructional objectives according to the curriculum requirements. | 1 | 2 | 3 | 4 | 5 | 6 |
| **1.3** | Teachers establish instructional objectives according to students’ needs. | 1 | 2 | 3 | 4 | 5 | 6 |
| **1.4** | Teachers help students understand the objectives. | 1 | 2 | 3 | 4 | 5 | 6 |
| **1.5** | Teachers should establish success criteria when plan language activities. | 1 | 2 | 3 | 4 | 5 | 6 |
| **1.6** | Teachers help students understand success criteria. | 1 | 2 | 3 | 4 | 5 | 6 |
| **1.7** | Teachers select appropriate assessment methods according to students’ needs. | 1 | 2 | 3 | 4 | 5 | 6 |
| **1.8** | Teachers collect evidence of learning through other teachers. | 1 | 2 | 3 | 4 | 5 | 6 |
| **1.9** | Teachers collect evidence of learning through students themselves. | 1 | 2 | 3 | 4 | 5 | 6 |
| **1.10** | Teachers collect evidence of learning through students’ peers. | 1 | 2 | 3 | 4 | 5 | 6 |
| **1.11** | Teachers collect evidence of learning through parents. | 1 | 2 | 3 | 4 | 5 | 6 |
| **1.12** | Teachers collect evidence of learning through classroom observation. | 1 | 2 | 3 | 4 | 5 | 6 |
| **1.13** | Teachers collect evidence of learning through oral questioning. |  |  |  |  |  |  |
| **1.14** | Teachers collect evidence of learning through conferences with students after class. | 1 | 2 | 3 | 4 | 5 | 6 |
| **1.15** | Teachers collect evidence of learning through student portfolios. | 1 | 2 | 3 | 4 | 5 | 6 |
| **1.16** | Teachers collect evidence of learning through self-assessment. | 1 | 2 | 3 | 4 | 5 | 6 |
| **1.17** | Teachers collect evidence of learning through peer assessment. | 1 | 2 | 3 | 4 | 5 | 6 |
| **1.18** | Teachers collect evidence of learning through students’ oral presentation (e.g., duty report). | 1 | 2 | 3 | 4 | 5 | 6 |
| **1.19** | Teachers collect evidence of learning through games. | 1 | 2 | 3 | 4 | 5 | 6 |
| **1.20** | Teachers collect evidence of learning through role play activities. | 1 | 2 | 3 | 4 | 5 | 6 |
| **1.21** | Teachers collect evidence of learning through classroom tests. |  |  |  |  |  |  |
| **1.22** | Teachers collect evidence of learning through diction. | 1 | 2 | 3 | 4 | 5 | 6 |
| **1.23** | Teachers collect evidence of learning through exercises. | 1 | 2 | 3 | 4 | 5 | 6 |
| **1.24** | Teachers elicit evidence of learning through reading aloud and reciting. | 1 | 2 | 3 | 4 | 5 | 6 |
| **1.25** | Teachers take account of language knowledge (e.g., vocabulary, grammar) when interpreting assessment data. |  |  |  |  |  |  |
| **1.26** | Teachers take account of language skills (listening, speaking, reading and writing) when interpreting assessment data. | 1 | 2 | 3 | 4 | 5 | 6 |
| **1.27** | Teachers take account of students’ approaches to learning when interpreting assessment data. | 1 | 2 | 3 | 4 | 5 | 6 |
| **1.28** | Teachers take account of students’ affective attitudes (e.g., interests, attitudes) when interpreting assessment data. | 1 | 2 | 3 | 4 | 5 | 6 |
| **1.29** | Teachers compare students’ current performance against the pre-set learning objectives when interpreting assessment data. | 1 | 2 | 3 | 4 | 5 | 6 |
| **1.30** | Teachers compare students’ current performance against their previous performance when interpreting assessment data. | 1 | 2 | 3 | 4 | 5 | 6 |
| **1.31** | Teachers check the trustworthiness of judgements when interpreting assessment data. | 1 | 2 | 3 | 4 | 5 | 6 |
| **1.32** | Teachers look for overall patterns of students’ learning when interpreting assessment data. | 1 | 2 | 3 | 4 | 5 | 6 |
| **1.33** | Teachers provide feedback to students about current achievement through scores and grades. | 1 | 2 | 3 | 4 | 5 | 6 |
| **1.34** | Teachers provide feedback to students about current achievement through written comments. | 1 | 2 | 3 | 4 | 5 | 6 |
| **1.35** | Teachers provide feedback to students about current achievement through oral comments. | 1 | 2 | 3 | 4 | 5 | 6 |
| **1.36** | Teachers communicate with parents about current achievement of students. | 1 | 2 | 3 | 4 | 5 | 6 |
| **1.37** | Teachers provide feedback to help students understand their strengths in learning. | 1 | 2 | 3 | 4 | 5 | 6 |
| **1.38** | Teachers provide feedback to help students understand their weaknesses in relation to the learning objectives. | 1 | 2 | 3 | 4 | 5 | 6 |
| **1.39** | Teachers provide feedback to help students develop error detection strategies. | 1 | 2 | 3 | 4 | 5 | 6 |
| **1.40** | Teachers provide feedback to help students find ways of solving problems they have in their learning. | 1 | 2 | 3 | 4 | 5 | 6 |
| **1.41** | Teachers provide feedback to help students understand what need to do to improve their work. | 1 | 2 | 3 | 4 | 5 | 6 |
| **1.42** | Teachers provide feedback to help students become self-regulated in their learning (e.g., to be aware of their learning objectives). | 1 | 2 | 3 | 4 | 5 | 6 |

**Your classroom-based assessment practices**

How often do you use the following strategies when assessing your students? Please indicate your situation by using the following rating scale:

1=Never 2=Very rarely 3=Rarely

4=Occasionally 5=Frequently 6=Always

| **2.1** | I identify instructional objectives when designing language activities. | 1 | 2 | 3 | 4 | 5 | 6 |
| --- | --- | --- | --- | --- | --- | --- | --- |
| **2.2** | I establish instructional objectives according to the curriculum requirements. | 1 | 2 | 3 | 4 | 5 | 6 |
| **2.3** | I establish instructional objectives according to students’ needs | 1 | 2 | 3 | 4 | 5 | 6 |
| **2.4** | I help students understand the objectives | 1 | 2 | 3 | 4 | 5 | 6 |
| **2.5** | I establish success criteria when plan language activities. | 1 | 2 | 3 | 4 | 5 | 6 |
| **2.6** | I help students understand success criteria. | 1 | 2 | 3 | 4 | 5 | 6 |
| **2.7** | I select appropriate assessment methods according to students’ needs. | 1 | 2 | 3 | 4 | 5 | 6 |
| **2.8** | I collect evidence of learning through other teachers. | 1 | 2 | 3 | 4 | 5 | 6 |
| **2.9** | I collect evidence of learning through students themselves. | 1 | 2 | 3 | 4 | 5 | 6 |
| **2.10** | I collect evidence of learning through students’ peers. | 1 | 2 | 3 | 4 | 5 | 6 |
| **2.11** | I collect evidence of learning through parents. | 1 | 2 | 3 | 4 | 5 | 6 |
| **2.12** | I collect evidence of learning through classroom observation. | 1 | 2 | 3 | 4 | 5 | 6 |
| **2.13** | I collect evidence of learning through oral questioning. |  |  |  |  |  |  |
| **2.14** | I collect evidence of learning through conferences with students after class. | 1 | 2 | 3 | 4 | 5 | 6 |
| **2.15** | I collect evidence of learning through student portfolios. | 1 | 2 | 3 | 4 | 5 | 6 |
| **2.16** | I collect evidence of learning through self-assessment. | 1 | 2 | 3 | 4 | 5 | 6 |
| **2.17** | I collect evidence of learning through peer assessment. | 1 | 2 | 3 | 4 | 5 | 6 |
| **2.18** | I collect evidence of learning through students’ oral presentation (e.g., duty report). | 1 | 2 | 3 | 4 | 5 | 6 |
| **2.19** | I collect evidence of learning through games. | 1 | 2 | 3 | 4 | 5 | 6 |
| **2.20** | I collect evidence of learning through role play activities. | 1 | 2 | 3 | 4 | 5 | 6 |
| **2.21** | I collect evidence of learning through classroom tests. |  |  |  |  |  |  |
| **2.22** | I collect evidence of learning through diction. | 1 | 2 | 3 | 4 | 5 | 6 |
| **2.23** | I collect evidence of learning through exercises. | 1 | 2 | 3 | 4 | 5 | 6 |
| **2.24** | I elicit evidence of learning through reading aloud and reciting. | 1 | 2 | 3 | 4 | 5 | 6 |
| **2.25** | I take account of language knowledge (e.g., vocabulary, grammar) when interpreting assessment data. |  |  |  |  |  |  |
| **2.26** | I take account of language skills (listening, speaking, reading and writing) when interpreting assessment data. | 1 | 2 | 3 | 4 | 5 | 6 |
| **2.27** | I take account of students’ approaches to learning when interpreting assessment data. | 1 | 2 | 3 | 4 | 5 | 6 |
| **2.28** | I take account of students’ affective attitudes (e.g., interests, attitudes) when interpreting assessment data. | 1 | 2 | 3 | 4 | 5 | 6 |
| **2.29** | I compare students’ current performance against the pre-set learning objectives when interpreting assessment data. | 1 | 2 | 3 | 4 | 5 | 6 |
| **2.30** | I compare students’ current performance against their previous performance when interpreting assessment data. | 1 | 2 | 3 | 4 | 5 | 6 |
| **2.31** | I check the trustworthiness of judgements when interpreting assessment data. | 1 | 2 | 3 | 4 | 5 | 6 |
| **2.32** | I look for overall patterns of students’ learning when interpreting assessment data. | 1 | 2 | 3 | 4 | 5 | 6 |
| **2.33** | I provide feedback to students about current achievement through scores and grades. | 1 | 2 | 3 | 4 | 5 | 6 |
| **2.34** | I provide feedback to students about current achievement through written comments. | 1 | 2 | 3 | 4 | 5 | 6 |
| **2.35** | Teachers provide feedback to students about current achievement through oral comments. | 1 | 2 | 3 | 4 | 5 | 6 |
| **2.36** | I communicate with parents about current achievement of students. | 1 | 2 | 3 | 4 | 5 | 6 |
| **2.37** | I provide feedback to help students understand their strengths in learning. | 1 | 2 | 3 | 4 | 5 | 6 |
| **2.38** | I provide feedback to help students understand their weaknesses in relation to the learning objectives. | 1 | 2 | 3 | 4 | 5 | 6 |
| **2.39** | I provide feedback to help students develop error detection strategies. | 1 | 2 | 3 | 4 | 5 | 6 |
| **2.40** | I provide feedback to help students find ways of solving problems they have in their learning. | 1 | 2 | 3 | 4 | 5 | 6 |
| **2.41** | I provide feedback to help students understand what need to do to improve their work. | 1 | 2 | 3 | 4 | 5 | 6 |
| **2.42** | I provide feedback to help students become self-regulated in their learning (e.g., to be aware of their learning objectives). | 1 | 2 | 3 | 4 | 5 | 6 |

**Demographic information**

1. Gender: □Male □Female
2. Age:

□under 20 years old □21-30 years old □31-40 years old □41-50 years old □over 50 years old

1. Educational qualification: □Below Bachelor □Bachelor □Master □ Doctoral
2. You have been teaching English at primary school level for:

□within 1 year □1-5 years □6-10 years □11-15 years □16-20 years □more than 20 years

1. You are now teaching in a: □stated funded primary school □private primary school
2. You are now teaching (*you may choose more than one*):

□Grade 1 □Grade 2 □Grade 3 □Grade 4 □Grade 5 □Grade 6

1. Number of English classes you are teaching: **________**
2. Total number of English lessons you are teaching per week: **__________**
3. Have you received any course or training in assessment? □Yes □No

**Appendix B. Correlations of teachers’ CBA beliefs and practices**

|  | Teachers’ CBA practices | | | | | |
| --- | --- | --- | --- | --- | --- | --- |
|  | Practice-PA | Practice-SAO | Practice-SIA | Practice-FAT | Practice-MPJ | Practice-PEF |
| ***Teachers’ CBA beliefs*** | |  |  |  |  |  |
| Belief-PA | .516** | .446** | .188** | .275** | .424** | .390** |
| Belief-SAO- | .366** | .447** | .305** | .296** | .362** | .390** |
| Belief-SIA | .384** | .453** | .420** | .263** | .368** | .439** |
| Belief-FAT | .337** | .418** | .230** | .475** | .388** | .425** |
| Belief-MPJ | .391** | .415** | .256** | .329** | .498** | .456** |
| Belief-PDF | .472** | .526** | .197** | .325** | .487** | .589** |
| Belief-PEF | .291** | .336** | .217** | .312** | .382** | .388** |

**= *p*<.01
